# A fungus (*Trametes pubescens*) resists cadmium toxicity by rewiring nitrogen metabolism and enhancing energy metabolism

**DOI:** 10.3389/fmicb.2022.1040579

**Published:** 2022-11-21

**Authors:** Jing Liu, Pengcheng Fu, Li Wang, Xiuying Lin, Naeimeh Enayatizamir

**Affiliations:** ^1^State Key Laboratory of Marine Resource Utilization in South China Sea, Hainan University, Haikou, China; ^2^School of Food Science and Engineering, Hainan University, Haikou, China; ^3^Department of Soil Science, Faculty of Agriculture, Shahid Chamran University of Ahvaz, Ahvaz, Iran

**Keywords:** *Trametes pubescens*, cadmium, transcriptome, metabolome, amino acid metabolism, nitrogen metabolism

## Abstract

As a primary goal, cadmium (Cd) is a heavy metal pollutant that is readily adsorbed and retained in rice, and it becomes a serious threat to food safety and human health. Fungi have attracted interest for their ability to remove heavy metals from the environment, although the underlying mechanisms of how fungi defend against Cd toxicity are still unclear. In this study, a Cd-resistant fungus *Trametes pubescens (T. pubescens)* was investigated. Pot experiments of rice seedlings colonized with *T. pubescens* showed that their coculture could significantly enhance rice seedling growth and reduce Cd accumulation in rice tissues. Furthermore, integrated transcriptomic and metabolomic analyses were used to explore how *T. pubescens* would reprogram its metabolic network against reactive oxygen species (ROS) caused by Cd toxicity. Based on multi-omic data mining results, we postulated that under Cd stress, *T. pubescens* was able to upregulate both the mitogen-activated protein kinase (MAPK) and phosphatidylinositol signaling pathways, which enhanced the nitrogen flow from amino acids metabolism through glutaminolysis to α-ketoglutarate (α-KG), one of the entering points of tricarboxylic acid (TCA) cycle within mitochondria; it thus increased the production of energy equivalents, adenosine triphosphate (ATP) and reduced nicotinamide adenine dinucleotide phosphate (NADPH) for *T. pubescens* to resist oxidative damage. This study can enable a better understanding of the metabolic rewiring of *T. pubescens under* Cd stress, and it can also provide a promising potential to prevent the rice paddy fields from Cd toxicity and enhance food safety.

## Introduction

Rapid technological development and urbanization, coupled with dramatic population growth, are posing significant impacts on the environment, ecosystems, and human society worldwide. As a result, farmland pollution is concerned as one of the most severe global issues that has prompted all countries in the world to make more efforts on the remediation of agricultural lands for environmental protection and sustainable development of the economy (Xie et al., 2019; Wei et al., 2020; Zhang et al., 2020).

Cadmium is a widely dispersed metal element that may be released into the environment with accelerated anthropogenic activities. This heavy metal is active in the migration between the soil–plant rhizosphere and is toxic to animals, plants, and humans (Wang et al., 2020a). The increase in modern industrial and agricultural production, in particular the use of pesticides and fertilizers, has caused an elevated Cd release into the environment (Taiwo et al., 2016). Moreover, Cd pollution is an irreversible process that can barely be remediated by ordinary soil microbiota. Once absorbed, Cd may remain toxic in ecosystems or human bodies for more than 20 years (Martelli et al., 2006). On the other hand, Cd is a highly mobile element, which is easily absorbed and accumulated by plants in cropland (Sarwar et al., 2017; Chen et al., 2018b). As a long-term impact, Cd may not only inhibit the yield and quality of agricultural crops seriously but also invade human bodies through the food chain to accumulate and cause damage to human health (Liu et al., 2013; Shan et al., 2020). Once Cd accumulates in human blood, it will gradually interfere with renal metabolism and bone formation *in vivo* (Chen et al., 2018a). Even at a low level, the long-term presence of Cd poses a serious threat to human beings (Yaghoubian et al., 2016; Mishra et al., 2018).

It has been found that some soil microorganisms have developed survival strategies in high Cd-contaminated environments, and they are even able to reduce Cd bioavailability (Limcharoensuk et al., 2015). In recent years, approaches have been used to suppress Cd accumulation in the rice grains in paddy fields (Lin et al., 2016). There are several approaches to microbial remediation for Cd pollution, including biosorption, biomineralization, biotransformation, and bioaccumulation (Ayangbenro and Babalola, 2017). Some researchers have explored rice contamination through the rhizosphere within a Cd-rich environment (Patten and Glick, 2002). Exogenous treatment with indole-3-acetic acid is seen to alleviate Cd accumulation in the shoots and roots of wheat seedlings (Agami and Mohamed, 2013). A Cd-resistant *Ochrobactrum* sp. capable of producing siderophore, ACC deaminase, and catalase is found to reduce the metal toxicity to rice (Pandey et al., 2013). It is well known that fungi are able to tolerate and detoxify heavy metal-contaminated effluents (Jacob et al., 2018). However, due to the high toxicity of Cd, so far, few fungi have been used as biological adsorbents to prevent the rice from Cd contamination.

*Trametes pubescens* MB 89 is a white-rot fungus capable of laccase production in the presence of copper ions (Tian et al., 2018). While it has strong resistance to heavy metals, *T. pubescens* has been reported for its use in wastewater treatment (Strong and Burgess, 2007, 2008). There are no studies so far on the removal of Cd from rice paddy fields. Furthermore, there are no reports yet on the molecular mechanisms for the detoxification of Cd toxicity in *T. pubescens*. We were involved in a practical project aiming at the prevention of cadmium pollution in the vast rice paddy fields to ensure food safety. We conducted research on the coculture of fungus and rice paddy first and found the effective avoidance of cadmium accumulation in rice with this technique. As a logical research paradigm shift, we then focused on the study of mechanisms regarding how the fungal cells reprogram their metabolism to reduce the cadmium ions for their detoxification. More specifically, *T. pubescens* was studied for its resistance to Cd toxicity and its promotion of rice growth with the elimination of Cd. Our specific objectives were: (i) to evaluate the effects of Cd on the growth of *T. pubescens* and its Cd adsorption; (ii) to investigate *T. pubescens* colonization on the rice growth and Cd removal from paddy tissue by pot hydroponic experiments; (iii) to integrate transcriptome and metabolome analysis to provide a better understanding of the mechanisms of tolerance to Cd toxicity in *T. pubescens.* This study provided valuable molecular information regarding the responses of *T. pubescens* to Cd contamination and revealed the metabolic pathways that play important roles in the tolerance of *T. pubescens* to Cd stress.

## Materials and methods

### Fungus cultivation and cadmium resistance

*Trametes pubescens* MB 89 (Netherlands, CBS 696.94) was obtained from Dr. Naeimeh Enayatizamir (Shahid Chamran University, Ahvaz, Iran). The spores of *T. pubescens* were inoculated in a sterilized liquid medium [10 g glucose, 20 g peptone from casein, 0.9 g (NH_4_)_2_SO_4_, 2 g KH_2_PO_4_, 0.5 g MgSO_4_7H_2_O, 0.1 g CaCl_2_2H_2_O, 0.5 g KCl, 0.5 g thiamine per liter of medium, and 20% citrate phosphate buffer solution, pH 4.5] in 28°C incubator for 15 days. The fungus was maintained on a potato dextrose agar plate at 4°C.

The Cd resistance of *T. pubescens* was investigated by monitoring its radial growth on potato dextrose agar (PDA) medium (1/4-Strength PDA) containing different concentrations of Cd^2+^ (0, 10, 25, 50, 100, and 200 mg/L). A plug of fungal inoculum was placed on a medium and incubated at 28°C for 6 days. Radial growth of *T. pubescens* was measured daily, and the inhibition percentage of fungal growth was evaluated.

### Cadmium adsorption capacity

The cadmium adsorption test was carried out in 250 ml Erlenmeyer flasks filled with 100 ml liquid medium with different concentrations of Cd^2+^ (0, 5, 10, 50, and 100 mg/L) addition on the 8th day of cultivation. The culture flasks were placed in an incubator at a constant temperature of 28°C. After adding Cd, flasks were moved from the incubator to a shaker to the culture at a constant rotation rate of 130 rpm. The cadmium adsorption test was conducted for another 7 days. The *T. pubescens* biomass was collected and freeze-dried for further study. After adsorption, the residual broth was examined for the Cd ion concentration in the solution. The Cd ions absorbed by the fungus were calculated *via* the following equation (Xu et al., 2012):


(1) $$q_{e}=\frac{V\times \left(C_{i}-C_{e}\right)}{W}$$

(2) $$Adsorptionrate\left(\%\right)=\frac{\left(C_{i}-C_{e}\right)}{C_{i}}\times 100\%$$

where q_*e*_ is the Cd uptake in mg Cd^2+^ kg^–1^ biomass; V is the value of the metal-containing solution in mL; Ci and Ce are the initial and equilibrium concentrations of Cd^2+^ in the solution, respectively, in mg L^–1^; and W is the weight of dry mycelia in g.

### Fourier transform infrared spectroscopy and scanning electron microscope analysis

The functional groups of fungal cell surface area before and after Cd^2+^ treatment were analyzed by Fourier transform infrared spectroscopy (FTIR, Perkin Elmer Frontier, Perkin Elmer Inc., Waltham, MA, USA) after freeze-drying. The KBr matrix (Sigma) and the scanning wavelength in the range of 500–4000 cm^–1^ were applied in this process. The scanning electron microscope (SEM, Hitachi S-4800 Hitachi, Ltd, Chiyoda-ku, Japan) was used to analyze the morphological structure and to evaluate the Cd adsorption.

### *Trametes pubescens* alleviated cadmium poisoning of rice

#### Construction of fungus-rice coculture system for prevention of cadmium accumulation in rice

The fungus-rice coculture system consisted of three parts: *T. pubescens* seed culture, plant samples preparation, and co-growth in the high concentration of Cd environment. The rice variety used in this study was Xiaonongzhan, and it was obtained from Jiangmen city in Guangdong Province, China. Seeds were sterilized with 5% H_2_O_2_ and germinated on filter paper at 25°C in the dark for 3–4 days. After germination, uniform length of plantlets was transferred into small trays with fixed plastic cups (4 cm diameter and 5 cm height, 12 plants per cup) and grown hydroponically in Hewitt nutrient medium (Liu et al., 2004) for 7 days for acclimatization. At this stage, the seedlings were placed in a light incubator (16 h light/8 h dark) at a constant temperature of 26°. *T. pubescens* was grown in a 1,000 ml Erlenmeyer flask containing 100 ml of the above-mentioned culture medium. They were statically incubated at 28°C, supplemented with 0.5 mM Cu^2+^ to stimulate laccase production on the third day of cultivation. After 10 days of cultivation, the spores and liquid solution were used as bioremediation agents.

Subsequently, 27 cups of rice were divided into three groups, namely, the control group (CONT), Cd treatment group [CONT(Cd)], and Cd plus *T. pubescens* group [Tp (Cd)]. For the Cd plus *T. pubescens* and Cd groups, the concentration of Cd^2+^ was 10 mg/L. Moreover, the prepared bioremediation agents were added to the Tp (Cd) group. The plants were grown in a tissue culture room at 25 ± 2°C with 14 h light/10 h dark and 60% relative humidity for 15 days.

#### Morphological observation of rice plants

After 15 days of cultivation, the plants were harvested, and the morphological observation of rice in different groups was carried out. The plants were washed with sterilized distilled water; then, the root and shoot length of the plants and root collar diameter were measured by a metric scale. In addition, plant development in terms of the number of leaves and branches was recorded. The paraffin section was used to observe the cell morphology of roots.

#### Cadmium accumulation in rice tissue quantification

Cadmium accumulation in rice root and the shoot was estimated as follows: after completion of 15 days, harvested plants were washed thoroughly with demineralized water, blotted, and oven-dried (80°C) for 1 day. Approximately, 0.2 g of dried powder of root and shoot was digested in 3 ml HNO_3_ at 120°C for 6 h (Awasthi et al., 2018). After digestion, the volume was constant at 50 ml by Milli-Q water. Cadmium accumulation in cellular shoot and root was quantified by atomic absorption spectrometry, respectively.

### Transcriptome profiling

Total RNA was extracted using an RNAprep Pure Plant Kit (Tiangen Biotech, Beijing, China) and then reverse transcribed into cDNA. Deep sequencing was then performed on Illumina NovaSeq (Illumina Inc., San Diego, CA, USA) at Majorbio Bio-Pharm Technology Co., Ltd. (Shanghai, China). Clean reads were mapped to the reference genome sequence of *T. pubescens* (GCA_001895945.1^1^). Only the reads with a perfect match or one mismatch were further analyzed and annotated, based on the reference genome. Hisat2 software was used for mapping with the reference genome. After the read counts of genes/transcripts were obtained by gene expression analysis, differentially expressed genes/transcripts were identified by using DESeq2 for multiple (≥2) samples or groups. Gene function was annotated based on the following databases: NCBI non-redundant protein sequences (NR), Swiss-Prot, protein family (Pfam), Clusters of Orthologous Groups of proteins (COG), Gene Ontology (GO), and Kyoto Encyclopedia of Genes and Genomes (KEGG). The *P*-values were adjusted to control the false discovery rate by using Benjamin and Hochberg’s methods, and *P* < 0.05 and log2 fold change value > 1 were assigned as the thresholds for differentially expressed genes. Enrichment analysis of the differentially expressed genes (DEGs) was analyzed using the GO and KEGG databases to obtain a detailed description of the DEGs. All the DEGs were analyzed using the R package from Bioconductor on the Majorbio Cloud Platform^2^.

### Metabolome profiling

Metabolites were extracted from a 50 mg sample using a 400 μl methanol: water (4:1, v/v) solution, with 0.02 mg/ml L-2-chlorophenylalanin as internal standard. The mixture was allowed to settle at –10°C and treated by a high throughput tissue crusher, Wonbio-96c (Shanghai Wanbo Biotechnology Co., Ltd.) at 50 Hz for 6 min, followed by ultrasound at 40 kHz for 30 min at 5°C. The samples were placed at –20°C for 30 min to precipitate proteins. After centrifugation at 13,000 *g* at 4°C for 15 min, the supernatant was carefully transferred to sample vials for ultra-high-performance chromatography (UHPLC)-MS/MS analysis. As a part of the system conditioning and quality control process, a pooled quality control sample (QC) was prepared by mixing equal volumes of all samples. Chromatographic separation of the metabolites was performed on a Thermo UHPLC system equipped with an ACQUITY UPLC HSS T3 (100 mm × 2.1 mm i.d., 1.8 μm; Waters, Milford, MA, USA). The mass spectrometric data were collected using a Thermo UHPLC-Q Exactive HF-X Mass Spectrometer equipped with an electrospray ionization source operating in either positive or negative ion mode.

After UHPLC-MS/MS analyses, the raw data were imported into the Progenesis QI 2.3 (Nonlinear Dynamics, Waters, USA) for peak detection and alignment. The preprocessing results generated a data matrix that consisted of the retention time (RT), mass-to-charge ratio (m/z) values, and peak intensity. Metabolic features detected with at least 80% in any set of samples were retained. After filtering, minimum metabolite values were imputed for specific samples in which the metabolite levels fell below the lower limit of quantitation and each metabolic feature was normalized by sum. A multivariate statistical analysis was performed using ropls (Version1.6.2^3^) R package from Bioconductor on Majorbio Cloud Platform^4^ (see footnote 2). Orthogonal partial least squares discriminate analysis (OPLS-DA) was used for statistical analysis to determine global metabolic changes between comparable groups. Variable importance in the projection (VIP) was calculated by the OPLS-DA model. *P*-values were estimated with paired Student’s *t*-test on single dimensional statistical analysis. Differential metabolites among the two groups were summarized and mapped into their biochemical pathways through metabolic enrichment and pathway analysis with the aid of a database search (KEGG^5^).

### Gene expression via qRT-PCR validation

Total RNA was extracted using an RNAprep Pure Plant Kit (Tiangen Biotech, Beijing, China) according to the manufacturer’s instructions. Total RNA of 200 ng/μl was reversely transcribed using a HiScript III All-in-one RT SuperMix Perfect for qPCR kit (Vazyme, China). The quantitative real-time PCR (qRT-PCR) was performed using a ChamQ Universal SYBR qPCR Master Mix kit (Vazyme, China) and ABI QuantStudio 6 Flex RT-PCR System (ABI, New York, NY, USA). The expression of target genes was normalized to the expression of *β-actin* and shown as a fold change relative to the control group based on the 2^–△^^△^ *^Ct^* method. The primer sequences are shown in Supplementary Table 1.

### Statistical analysis

All the results were expressed as the mean and standard deviation. For multiple group comparison, one-way analysis of variance (ANOVA) followed by Tukey’s *post hoc* test (SPSS26, Inc., Chicago, IL, USA) was performed to identify differences among means. Statistical significance is represented by *p < 0.05, **p < 0.01, ***p < 0.001. Sample size and statistical tests are also indicated in the figure legends.

## Results

### The response to cadmium stress and cadmium removal capacity of *Trametes pubescens*

After 7 days of cultivation, the fungal growth (in PDA medium), biosorption capability, Cd removal efficiency, SEM, and FTIR were measured to evaluate the performance of *T. pubescens* under different Cd stress conditions.

**FIGURE 1:**
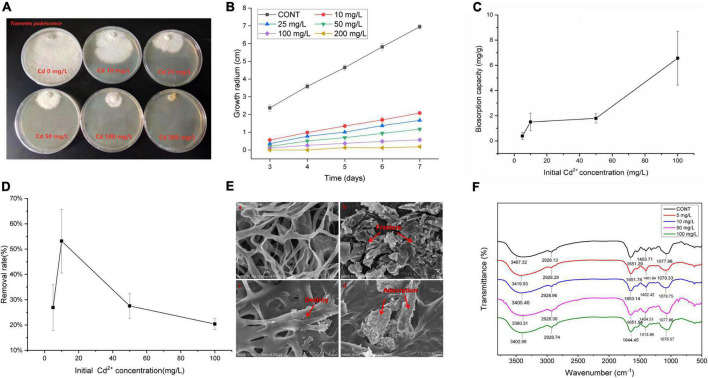
Effects of cadmium exposure in *T. pubescens* and its cadmium removal capacity. **(A)** *T. pubescens* growth on PDA amended with different concentrations of Cd^2+^ for 7 days. **(B)** Growth inhibition of *T. pubescens* with varying Cd^2+^ concentrations (0–200 mg/L). **(C)** Biosorption capacity of *T. pubescens* in different concentrations of Cd^2+^ for 7 days exposure. **(D)** The removal rate of *T. pubescens* in different concentrations of Cd^2+^ for 7 days exposure. **(E)** SEM micrograph of *T. pubescens* biomass under Cd^2+^ stress (a): without Cd^2+^ treatment (CONT); (b): treated with 100 mg/L of Cd^2+^; (c,d): treated with 10 mg/L of Cd^2+^ after 7 days exposure. **(F)** FTIR spectra of *T. pubescens* grown for 7 days in a Cd-free medium and the presence of Cd ions (initial concentration: 0 mg/L, 5 mg/L, 10 mg/L, 50 mg/L, 100 mg/L). CONT, no Cd treatment group.

The radius growth diameter of the strain in the PDA plate was measured every 24 h with a different concentration gradient of Cd^2+^, as shown in Figures 1A,B. It can be seen that *T. pubescens* grew well at the Cd^2+^ concentration of 10 mg/L. With the increase of Cd concentration, the growth of *T. pubescens* was gradually inhibited. When Cd^2+^ concentration reached 200 mg/L, there was no hypha observed. The Cd removal by *T. pubescens* from the aqueous solution results indicated that with the initial Cd^2+^ concentration of 100 mg/L, the Cd adsorption of viable *T. pubescens* mycelium was 6.565 mg/g which was the highest in this study (Figure 1C). The maximum removal rate of *T. pubescens* was observed to be 53.13% when the initial Cd concentration was 10 mg/L (Figure 1D).

Scanning electron microscope observations indicated that a large number of mycelia were deformed and became fragmented when the Cd concentration in the medium was too high (100 mg/L Cd^2+^) for the fungus to bear (Figure 1Eb). It was seen that the mycelia in the shaking flasks initially grew together, and then the aggregation eventually fell apart. Nevertheless, dense nanoscale particles on the cell surface of mycelium were observed in the low Cd experimental groups (10 mg/L Cd^2+^) (Figure 1Ed), while these particles were not seen in the control group (Figure 1Ea). It is postulated that the dense nanoparticles were formed after *T. pubescens* had adsorbed Cd and been complex sediments formation. In low concentrations of Cd, Cd seemed to still be able to deform the mycelia (Figure 1Ec).

The FTIR analysis was implemented to verify the metal ion interacting with the functional groups existing on the fungal surface in the wavelength of 500–4,000 cm^–1^. The FTIR spectra of *T. pubescens* exposed to Cd showed varying asymmetrical stretching bands and peaks in Figure 1F. Among them, the stretching vibration peaks of amino and hydroxyl groups were found to shift from 3487.32 cm^–1^ (control group) to 3419.93 cm^–1^ (5 mg/L Cd), 3405.40 cm^–1^ (10 mg/L Cd), 3390.31 cm^–1^ (50 mg/L Cd), and 3402.99 cm^–1^ (100 mg/L Cd), indicating that these hydroxyl groups and amino groups from a polysaccharide, fatty acid, and protein components participated in the adsorption process (Zhou et al., 2016). In addition, there is a C–H stretching vibration peak near 2,928 cm^–1^, and the slight shift of the spectrum after adsorption indicates that the C–H of methyl groups participated in the Cd adsorption process (Khan et al., 2018). The carbonyl stretching vibration of amide and –NH distortion bands was observed at 1,651 cm^–1^ (Nagesh et al., 2015). The spectrum shows the low-intensity vibration deviation of band 1077 cm^–1^, and the peak that shifted from 1084 cm^–1^ to 1075 cm^–1^ could be attributed to the C–O stretching of carboxyl groups and S = O groups (Loukidou et al., 2003).

### *Trametes pubescens* stimulated rice growth, protected root cells from disrupture, and reduced tissue cadmium accumulation under cadmium stress

**FIGURE 2:**
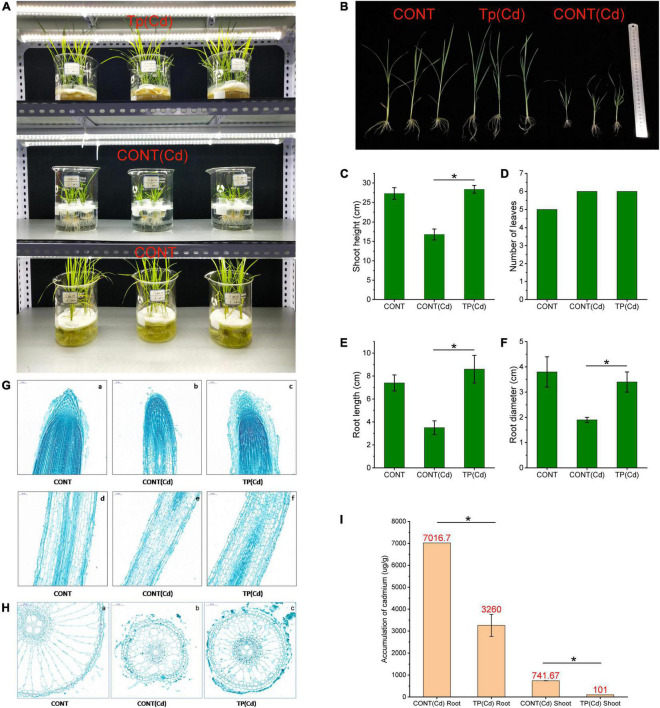
Effect of *T. pubescens* colonization on rice seedlings under Cd stress. **(A)** Fungus-rice coculture model constructed in the laboratory. **(B)** The effect of *T. pubescens* colonization on the growth of rice seedlings under Cd stress. **(C)** Shoot height of rice seedlings. **(D)** Leave number of rice seedlings. **(E)** Root length of rice seedlings. **(F)** Root diameter of rice seedlings. **(G,H)** Paraffin section of rice root tissues under Cd stress, (a–c) longitudinal section of root tip and (d–f) cross section of root. **(I)** Accumulation of Cd in root and shoot of rice after 15 days culture. * Indicates significant differences between groups (*p* < 0.05). Cd, cadmium; CONT, no Cd treatment group; CONT(Cd), Cd treatment and without *T. pubescens* colonization group; Tp (Cd), Cd treatment and with *T. pubescens* colonization group.

In order to explore the application potential of *T. pubescens*, we established a coculture system for both fungi and rice seedlings to investigate the effect of fungi on Cd removal in rice. Rice seedlings were grown in a plant tissue culture chamber exposed to a specific temperature, humidity, and light conditions. Cd stress experiments were performed by adding CdCl_2_ to a final concentration of 10 mg/L. Under Cd stress, it was found that the rice seedling growth was significantly inhibited, which was characterized by short plant height and yellow withered leaves (Figures 2A,B). However, there was no remarkable difference between the growth of rice seedlings colonized with *T. pubescens* and the CONT(Cd) group. Further measurement results (Figures 2C–F) indicated that the addition of Cd^2+^ had significantly inhibited the growth of rice seedlings in both the height of shoots and the length/diameter of root decreased. It was found that the shoot height was almost 38.46% less in Cd^2+^-treated seedlings than in non-treated ones, while the root length was almost 52.5% less in Cd^2+^-treated seedlings than in the control group. Furthermore, the root diameter was almost 49.2% less in Cd^2+^-treated seedlings than in non-treated seedlings.

Evidence has suggested that active growth of the roots accelerated the absorption of nutrients and thereby facilitated shoot growth (Cai et al., 2020). It is seen from the vertical section of rice roots (Figure 2G) that the CONT(Cd) with Cd showed retarded plant development with cell degeneration of the root tips and root thinning. Moreover, in Figure 2H (with root cross-section slices), Cd contents in rice plants have caused serious damage to the cell morphology of roots and further resulted in cell deformation and senescence. The *T. pubescens* colonization has protected root cells from disruptors. As a result of Cd adsorption by *T. pubescens*, the residual Cd concentration in the medium was significantly reduced, which alleviated the toxicity of Cd to roots. Furthermore, Cd ion might degrade root tip cells and switch down water absorption and transport system, thus resulting in reduced nutrient supply. In comparison, the development of the root tips for the rice plants in the Tp (Cd) group was healthy and similar to that for the CONT group. As can be seen from the comparison between Figures 2Gb and 2Gc, *T. pubescens* colonization on rice plants could significantly prevent the rice roots from the damage by environmental Cd, protect the rice root tip cells, and enable absorption and transportation of nutrients from the rhizosphere microenvironment (Moon et al., 2019). The results suggested that *T. pubescens* has played an important role in protecting rice plants from Cd-induced damage.

Regardless of the presence or absence of soil microorganisms, roots usually accumulated more Cd than that culms, leaves, and grains (Shan et al., 2020). After 15 days of growth in an aqueous culture medium with 10 mg/ml Cd, the Cd accumulation in the roots and shoots of the rice plants is shown in Figure 2I. For seedlings colonized by *T. pubescens*, Cd concentrations in roots and shoots were reduced by 53.54 and 86.38%, respectively. These results clearly showed that *T. pubescens* was able to suppress the accumulation of Cd in rice. It, thus, has a positive potential for heavy metal removal for more sustainable agriculture.

### Specific differentially expressed genes of *Trametes pubescens* under cadmium treatment and transcriptome analysis

**FIGURE 3:**
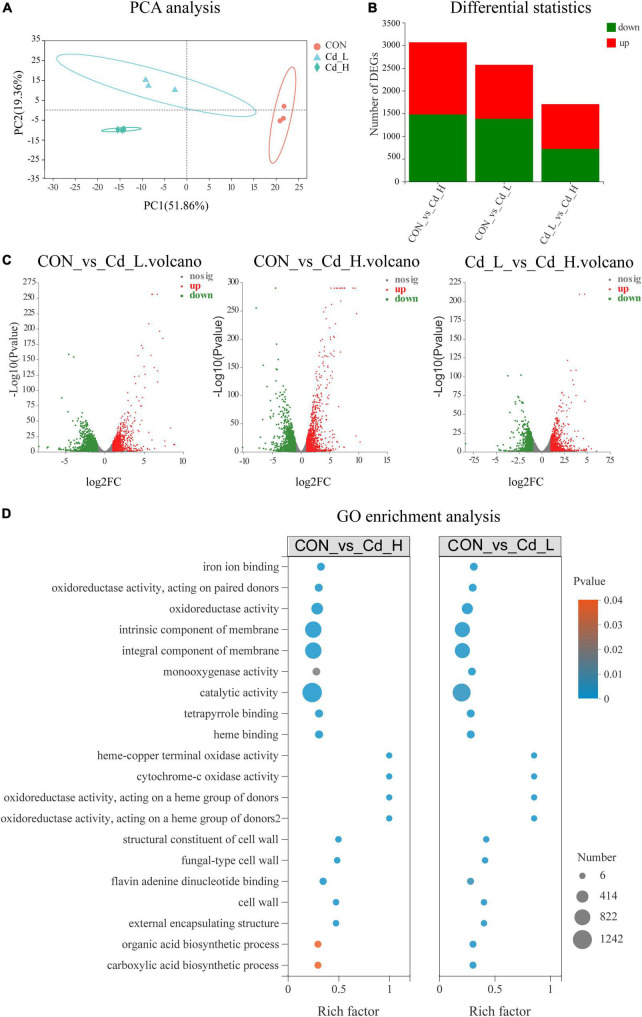
Effects of Cd stress on *T. pubescens* by transcriptomic analysis. **(A)** PCA analysis of the three groups (Cd_L, Cd_H, CON). **(B)** Statistics of DEGs. **(C)** Volcano plot (based on the combination of positive and negative ions) between groups. **(D)** GO enrichment analysis between groups. Cd, cadmium; Cd_L, low-dose Cd treatment group; Cd_H, high-dose Cd treatment group; CON, no Cd treatment group; PCA, principal components analysis; DEGs, differentially expressed genes; GO, Gene Ontology.

To further investigate how the fungi defend against Cd toxicity and the adaptive performance of *T. pubescens* under low/high Cd concentration, three groups: Cd_L (*T. pubescens* in low Cd concentration, 5 mg/L), Cd_H (*T. pubescens* in high Cd concentration, 10 mg/L), and CON (without Cd stress, 0 mg/L) were organized, and the samples on the 15th day’s culture were subjected to RNA-Seq analyses to demonstrate the potential resistance of *T. pubescens* against Cd toxicity. In the transcriptome analysis, a total number of 14,442 non-redundant transcripts were annotated in GO, KEGG, COG, NR, Swiss-Prot, and Pfam databases, with 9442, 4462, 1405, 14442, 6589, and 8251 corresponding annotated unigenes (Supplementary Figure 1). The principal component analysis (PCA) showed that the expressed genes of *T. pubescens* under different Cd concentration treatments were profoundly discrepant (Figure 3A). The DEGs were screened between groups (CON vs. Cd_L, CON vs. Cd_H, Cd_L vs. Cd_H), and a total of 2572, 3067, and 1705 DEGs were identified (Figure 3B). The volcano plot represented the multiples of expression differences and the statistical test values of gene expression changes between the two groups (Figure 3C). It was clearly seen that there was a significant change in gene expression levels between CON and Cd_H.

To explore the possible functions of DEGs, cluster analysis on the expression patterns of the fungal gene sets was performed, with the aid of heat maps and subcluster trend maps (Supplementary Figure 2). From the figure, it was seen that the genes with similar expression patterns were functionally related to groups with high consistency, which ensured the reliability of the subsequent analysis. GO annotations analysis of DEGs showed the main GO terms related to the “biological process” were metabolic process, cellular process, and biological regulation, while the more abundantly annotated DEG focused on membrane components, intracellular organelles, and protein-containing complexes for catalytic activity, binding, and transporter activity (Supplementary Figure 3). Moreover, DEGs were annotated using the KEGG database and classified according to the pathways or functions they participated (Supplementary Figure 4). The transcriptome results indicated that the most abundantly annotated KEGG pathways were carbohydrate metabolism, amino acid metabolism, and ABC transporters for the membrane. In addition, it was observed that signal transduction by the MAPK signaling pathway and phosphatidylinositol signaling system was significantly (*P* < 0.05) upregulated under Cd stress.

**FIGURE 4:**
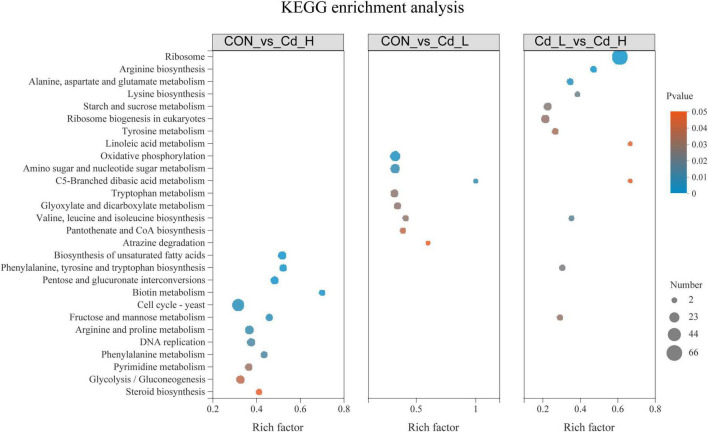
Kyoto encyclopedia of genes and genomes (KEGG) pathway enrichment analysis between different Cd stress groups in the transcriptome. Cd, cadmium; Cd_L, low-dose Cd treatment group; Cd_H, high-dose Cd treatment group; CON, no Cd treatment group; KEGG, Kyoto Encyclopedia of Genes and Genomes.

To further investigate the Cd stress-adaptive mechanism of *T. pubescens*, GO enrichment and KEGG enrichment of DEGs were further analyzed using Goatools software. GO enrichment analysis of differential expression of the “CON vs. Cd_L” set and “CON vs. Cd_H” set revealed that the response of *T. pubescens* to Cd toxicity (Figure 3D) involved metabolic reprogramming in catalytic activity, intrinsic/integral component of membrane, oxidoreductase activity, iron ion/tetrapyrrole/heme/flavin adenine dinucleotide binding, monooxygenase/heme–copper terminal oxidase/cytochrome-c oxidase activity, and cell wall functions. Meanwhile, the KEGG enrichment analysis (Figure 4) also showed that the DEGs appeared in genetic information processing ribosome and non-homologous end-joining; biotin metabolism; amino acid metabolism (phenylalanine, tyrosine, and tryptophan biosynthesis; arginine and proline metabolism; arginine biosynthesis; tryptophan metabolism); carbohydrate metabolism (C5-branched dibasic acid metabolism; amino sugar and nucleotide sugar metabolism; pentose and glucuronate interconversions); and biosynthesis of unsaturated fatty acids.

### Metabolome analysis of *Trametes pubescens* responsive to cadmium toxicity

**FIGURE 5:**
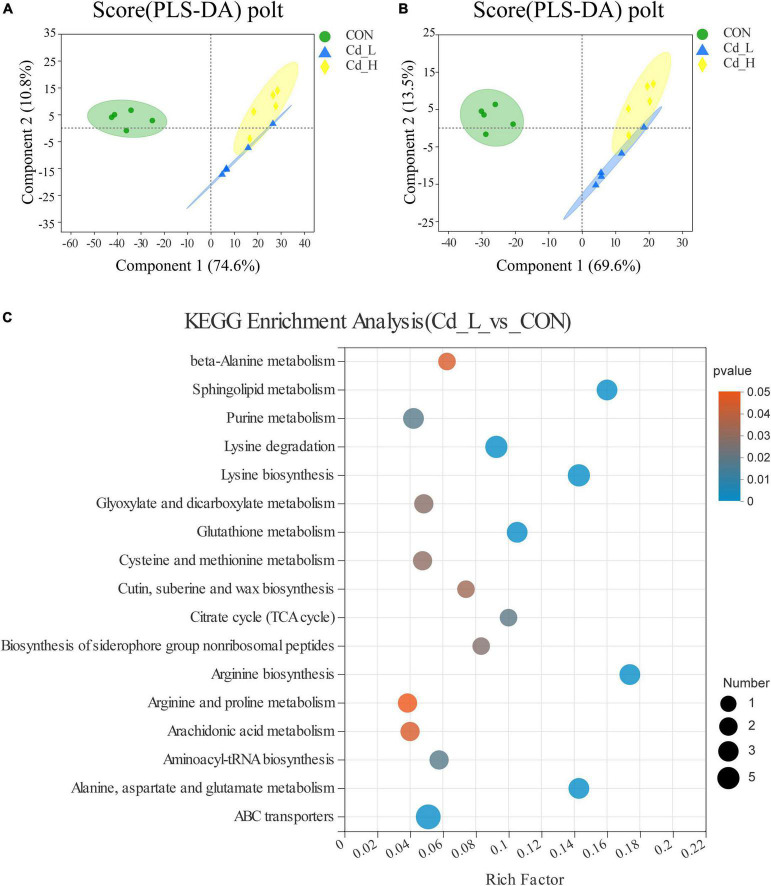
Effects of Cd stress on *T. pubescens* metabonomic profiling by UHPLC-Q Exactive MS. **(A,B)** PLS-DA score plot of three groups (Cd_L, Cd_H, CON) in the positive ion and negative ion. **(C)** KEGG enrichment analysis between Cd_L and CON groups. Cd, cadmium; Cd_L, low-dose Cd treatment group; Cd_H, high-dose Cd treatment group; CON, no Cd treatment group; UHPLC, ultra-high-performance liquid chromatography; PLS-DA, partial least squares discriminant analysis; KEGG, Kyoto Encyclopedia of Genes and Genomes.

To explore the metabolic response of *T. pubescens* under Cd stress, the fungal metabolic profiles were performed by UHPLC-MS/MS. A total of 10,462 peaks (5958 ESI + ions and 4504 ESI- ions) were detected, and 726 metabolites were identified in all *T. pubescens* samples. The partial least squares discriminant analysis (PLS-DA) showed that the metabolic performance of *T. pubescens* under Cd treatment was significantly distinguishable from the control group (Figures 5A,B). The differentially expressed metabolites (DEMs) were annotated and classified into 21 taxonomies according to the Human Metabolome Database (HMDB) (Supplementary Figure 5A), while “amino acids, peptides, and analogs” accounting for 19.76% were the highest. It was followed by “carbohydrates and carbohydrate conjugates” for 5.03% and “fatty acids and conjugates” for 4.33%. The KEGG pathway enrichment analysis of the DEMs between the Cd_L group and CON group (Cd_L_vs._CON) showed that amino acid metabolism (such as arginine and proline metabolism, beta-alanine metabolism, cysteine and methionine metabolism, glutathione metabolism, alanine, aspartate, and glutamate metabolism, lysine degradation, arginine biosynthesis, and lysine biosynthesis), lipid metabolism (arachidonic acid metabolism, cutin, suberine, and wax biosynthesis, and sphingolipid metabolism), carbohydrate metabolism (glyoxylate and dicarboxylate metabolism, citrate cycle), ABC transporters, purine metabolism, and aminoacyl-tRNA biosynthesis were significantly changed under 5 mg/L Cd stress (Figure 5C). As far as KEGG pathway enrichment analysis of the DEMs between the Cd_H group and CON group (Cd_H_vs._CON) (Supplementary Figure 5B), pantothenate and CoA biosynthesis, linoleic acid metabolism, and glycerophospholipid metabolism were significantly different. The KEGG pathway enriched between the Cd_L group and Cd_H group (Cd_L_vs._Cd_H) (Supplementary Figure 5C) was ABC transporters, amino sugar, and nucleotide sugar metabolism, aminoacyl-tRNA biosynthesis, ether lipid metabolism, fructose and mannose metabolism, glycerophospholipid metabolism, pentose phosphate pathway, purine metabolism, sphingolipid metabolism.

### Integrative analysis of the transcriptome and metabolome reveals the underlying mechanism of the response of *Trametes pubescens* to cadmium toxicity

**FIGURE 6:**
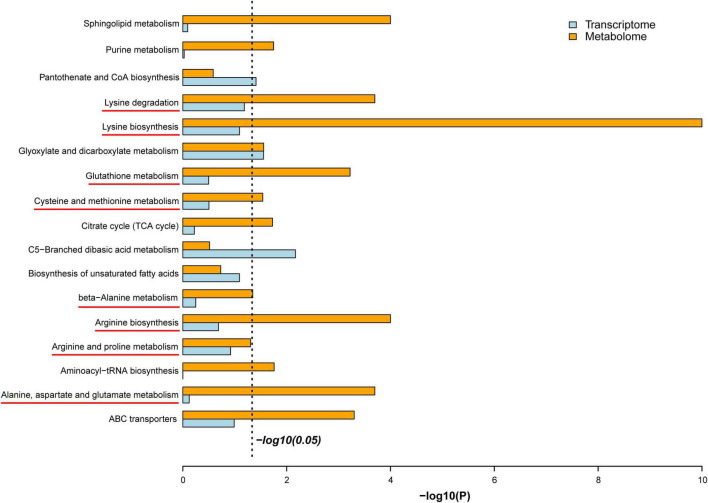
Integrative analysis of transcriptome and metabolome.

**FIGURE 7:**
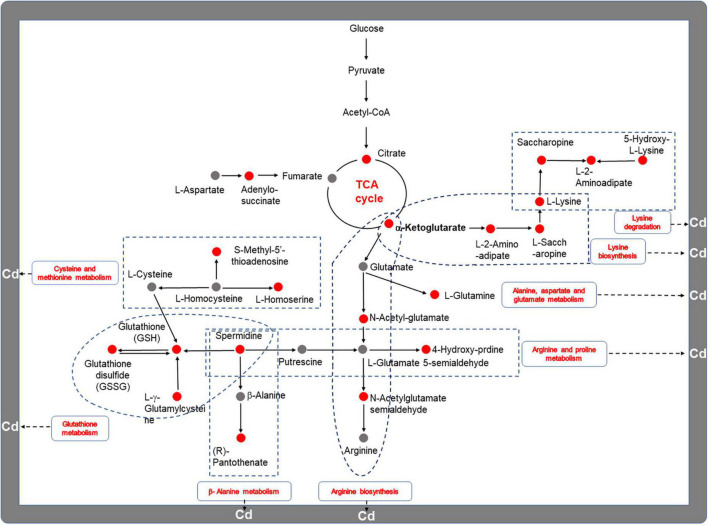
The underlying mechanism of *T. pubescens* defenses against Cd toxicity. The red color represents upregulated metabolites or metabolic pathways. The gray color indicates the metabolites that were not annotated. Cd, cadmium; Cd_L, low-dose Cd treatment group; CON, no Cd treatment group.

Based on the multi-omic analysis, we postulated that *T. pubescens* resists Cd toxicity by modulating the synthesis of non-essential amino acids (NEAAs) and rewiring nitrogen flow pathways to enhance cellular energy metabolism. From the integrative analysis of the DEGs (transcriptome) and the DEMs (metabolome) shown in Figure 6, it was found that *T. pubescens* resists oxidative stress caused by Cd stress by altering the nitrogen flow from amino acids metabolism through glutaminolysis to α-KG, one of the entering points of TCA cycle within mitochondria, and it, thus, increased the production of energy equivalents, ATP and NADPH for cellular survival. Amino acids are the predominant sources of nitrogen in cells; interestingly, we found that all the DEMs-related amino acid metabolism in both the Cd_L group and Cd_H group was significantly increased compared with the CON group (Supplementary Figure 6). NEAAs provide building blocks for protein and nucleotide synthesis, constitute components for redox homeostasis, one-carbon metabolism, and the urea cycle, and serve as important substrates for many nitrogenous compounds (Kurmi and Haigis, 2020). This evidence also indicates that the enhancement of NEAAs metabolism plays a vital function in the adaptation of *T. pubescens* to Cd stress. 2-Oxo-glutarate (α-ketoglutarate) is an intermediate of the TCA cycle which also be generated by oxidative deamination of glutamate (glutaminolysis) by the action of mitochondrial glutamic dehydrogenase. Fungal cells rewire nitrogen metabolism to enhance the TCA cycle and electron transport chain for more ATP and NADPH generation. In this way, the fungi can produce more energy to protect themselves against oxidative damage. In summary, the underlying mechanism of *T. pubescens* defends against Cd toxicity is illustrated in Figure 7. Previous reports have shown the significant beneficial functions of nitrogen metabolism in plant responses and adaptation to heavy metal stress (Sharma and Dietz, 2006). In this study, for the first time, we elucidated the reprogramming of nitrogen metabolism in fungi to resist Cd toxicity.

### Validation of differentially expressed genes results by qRT-PCR

**FIGURE 8:**
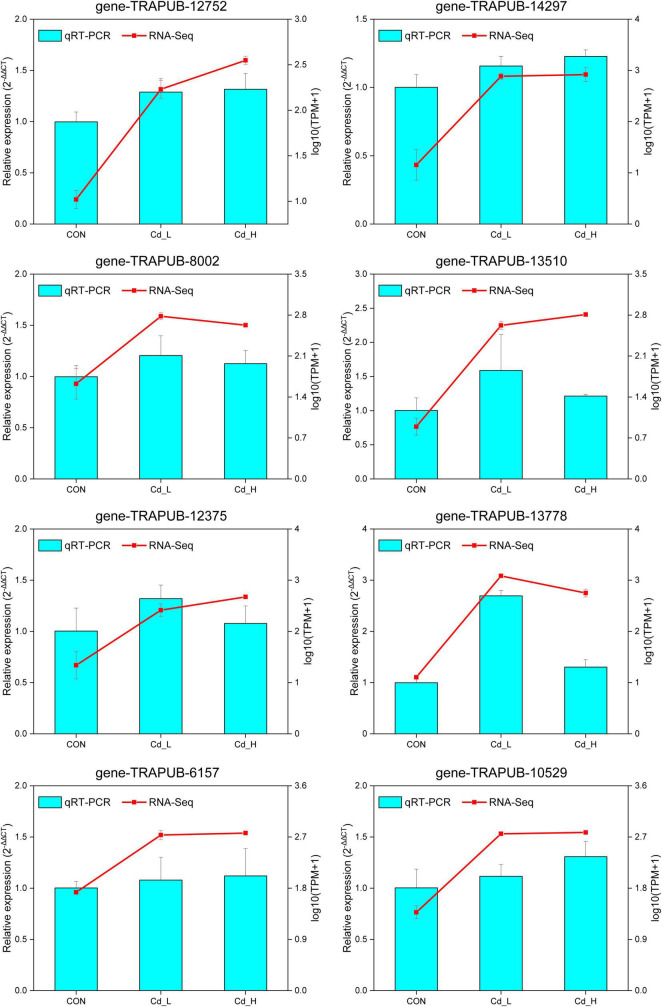
qRT-PCR verification of the DEGs in *T. pubescens* under different concentrations of Cd^2+^ stress. Comparison of the relative expression levels determined *via* qRT-PCR and RNA-Seq. Eight genes were selected, including gene-TRAPUB-12752, 14297, 8002, 13510, 12375, 13778, 6157, and 10529. The qPCR expression levels were calculated as a ratio relative to the level of expression in the group of CON, which was set as 1. All data indicate the mean ± SE, with three biological replications and three technical replications. Cd_L, low-dose Cd treatment group; Cd_H, high-dose Cd treatment group; CON, no Cd treatment group; qRT-PCR, real-time quantitative PCR; DEGs, differentially expressed genes.

To verify the accuracy and reproducibility of the transcriptome data, 8 representative DEGs were selected, and their expression levels were examined by qRT-PCR. The gene expression trends in the qRT-PCR analysis were in accordance with the fold change values shown in the transcriptome analysis (Figure 8). The results not only indicated that the transcriptome data were reliable but also were conducive to further verifying the roles of the responsible metabolic genes in response to Cd stress.

## Discussion

Strategies adopted by fungi for the remediation of heavy metals (Cd^2+^, Pb^2+^, Ni^2+^, Cu^2^+, etc.) in soil or water are associated with a two-step process: biosorption and bioaccumulation (Geetha et al., 2021; Priyadarshini et al., 2021). However, the in-depth explorations of underlying molecular mechanisms responsible for these processes are not fruitful yet. In this study, we evaluated the white-rot fungus *T. pubescens* with outstanding Cd tolerance and removal capacity, using a coculture system of rice seedlings and *T. pubescens.* It was demonstrated that the biological Cd detoxification in rice using the fungus *T. pubescens* is a promising and feasible approach. Furthermore, integrated transcriptome and metabolome profiling was used to explore the adaptive mechanism of *T. pubescens* under Cd Stress. It was found that the membrane transport, MAPK signaling pathway, and phosphatidylinositol signaling system were significantly changed to trigger the increase of nitrogen metabolism and energy metabolism for *T. pubescens* to tolerate Cd toxicity.

In the present study, the Cd tolerance of *T. pubescens* was up to 100 mg/L and the Cd adsorption of viable mycelium was 6.565 mg/g, which is four orders of magnitude higher than the maximum permissible limit for Cd in drinking water, which is 0.003 mg/L, stipulated by the World Health Organization (Priyadarshini et al., 2021). It has been reported that fungi have several strategies to survive metal stress (Zhao et al., 2016; Wang et al., 2019). In our study, the living *T. pubescens* mycelium with high Cd adsorption capacity makes it a potential biological agent to remove Cd^2+^ from the polluted environment. The removal rate from the medium was 53.13% with 10 mg/L initial Cd^2+^ concentration, which was higher than the study in the literature in which the removal efficiency of *A. aculeatus* reached the maximum (46.8%) at 10 mg/L Cd^2+^ concentrations (Xie et al., 2019). Our results confirmed that *T. pubescens* is able to remove Cd contamination.

The mechanisms utilized by the fungus to survive in the presence of metals includes binding metal ions to high-affinity functional groups, complexation with different microbial extracellular polymer, and metal accumulation within the cells (Geetha et al., 2021). SEM micrograph of the Cd-treated *T. pubescens* cells illustrated that Cd has caused certain toxicity and destruction to mycelium, while mycelium has extracellular adsorption and fixation effect on Cd to reduce its toxicity. The phenomenon is similar to the work with *Pseudomonas chengduensis* strain MBR in the previous study (Wang et al., 2020b). Jacob et al. (2017) reported the alteration of cell wall morphology due to heavy metal stress. It confirmed that heavy metals were able to inhibit various physiological processes such as cell membrane distribution and cell division (Khan et al., 2009; Yuan et al., 2015; Karthik et al., 2016), to depress enzyme activity, and to denature the proteins (Khan et al., 2009; Wyszkowska et al., 2014). Typically, high contents of carboxyl groups and the mannuronic and guluronic acids of the cell wall polysaccharides could enhance heavy metal biosorption (Raja et al., 2016). From the FTIR results, it is inferred that the functional groups of –OH, –NH, C-H, C = O, C-O, S = O exist on the surface of *T. pubescens* which was postulated to participate in the Cd^2+^ biosorption process.

The fungal detoxification of heavy metal in the contaminated environment includes valence transformation, intracellular and extracellular precipitations as well as active uptake (Thatoi et al., 2014). In this study, we were surprised to find that the colonization of *T. pubescens* in the rice seedlings had remarkably reduced the Cd toxicity and promoted the growth of rice paddies. Moreover, *T. pubescens* was also seen to reduce Cd accumulation in the roots and shoots of rice seedlings (Figure 2). The experiments proved that *T. pubescens* is a beneficial fungus that is able to effectively alleviate Cd stress in Xiaonongzhan rice.

Through the observation and analysis of the above experiments, we found that *T. pubescens* is a powerful fungus that can tolerate high concentrations of Cd, and it is a valuable microbial agent that effectively reduces Cd pollution in rice planting. We have then conducted in-depth studies on its response to Cd stress, particularly the changes in its metabolic pathway utilization in a Cd-polluted environment. Previous studies demonstrated that the defense mechanism to environmental stressors by the fungi included the transcriptional regulation of functional genes, activation of stress-related metabolic pathways (e.g., amino acid metabolism, energy metabolism), synthesis of biological substances (e.g., antioxidant enzymes, cellular amino acid, biostimulant, glutathione), and processing of environmental information (e.g., MAPK signaling pathway and membrane transport) (Wu et al., 2007), while the transcriptomic data indicated that there were 2572 and 3067 DEGs which were differentially expressed in 5.0 mg/ml and 10.0 mg/ml of CdCl_2_ medium, respectively, compared with the untreated group. Therefore, *T. pubescens* seems to be able to robustly alter its gene expression as a response to the stress triggered by Cd exposure. GO enrichment analysis showed that it is related to catalytic activity, component of membrane, oxidoreductase activity, iron ion/tetrapyrrole/heme/flavin adenine dinucleotide binding, and cell wall functions. KEGG analysis revealed that genetic information processing replication, repair, and translation were significantly regulated under Cd stress; moreover, metabolism of cofactors and vitamins, carbohydrate and amino acid, and biosynthesis of unsaturated fatty acids was altered to respond to the Cd stress.

Cadmium detoxification in soil and water has attracted widespread attention due to its ecological risk in water resources and cultivated lands (Yang et al., 2018). It was found that Cd could cause pathological tissue injury by inducing ROS generation and epigenetic changes in gene expression level (Wang et al., 2012). The GO “catalytic activity” was more abundant in the Cd_L group which indicated that *T. pubescens* produced more antioxidants to scavenge intracellular ROS and protect cells against heavy metals (Zhang et al., 2015). Meanwhile, oxidoreductase activity was also upregulated in response to the Cd presence as a defense mechanism against ROS production (Oshiquiri et al., 2020). One of the most important oxidoreductase glutathione S-transferases (GST) has been demonstrated to play a vital role in Cd detoxification (Yang et al., 2011). The “component of membrane” and “cell wall functions” were enrichment indicating that Cd can cause damage to the cell membrane or cellular structure (Xu et al., 2021). Heavy metal ions, like Cd, As, Pb, Hg, etc., were reported to acidify the cytoplasm and damage the cell membrane by disrupting its potential (Conner and Schmid, 2003; Samadani et al., 2018). In particular, Cd^2+^ could be adsorbed by the negatively charged functional groups available on the cell wall, while Cd^2+^ in excess could also enter the cells and adversely affect the viability of cells. Previous studies described that Cd-mediated inhibition of DNA repair mechanisms and apoptosis led to the accumulation of cells with unrepaired DNA damage, which in turn resulted in genomic instability (Filipiè, 2012). We observed that genetic information processing, DNA replication, and repair pathway were simultaneously upregulated in *T. pubescens*, suggesting that this fungus reprogrammed its metabolism in response to the DNA damage caused by heavy metal Cd^2+^. KEGG analysis revealed that multiple overexpressed genes have encoded its key enzymes involved in the metabolism of cofactors and vitamins, carbohydrate and amino acid, and biosynthesis of unsaturated fatty acids pathway to enable *T. pubescens* to adapt to Cd stress.

Cadmium-related effects on cellular comprises altered metabolism in plants and microorganisms. At the same time, some organisms with the adaptation of Cd^2+^ ions were able to alleviate the toxicity by regulating their metabolic pathways and metabolites in a short/long time and survived. It is interesting to explore this metabolic regulation process. In this study, the metabolome results showed that rewiring nitrogen metabolism has positively dominated the adaptation of *T. pubescens* against Cd toxicity, followed by carbohydrate metabolism and lipid metabolism. Differential gene expressions for the altered utilization of nitrogen metabolic pathways were seen to be a central response to heavy metals (Hussain et al., 2020). Upon exposure to metals, amino acids were found to be synthesized as radical scavengers and regulation of ion transport (Pavlíková et al., 2014). Amino acids rich in carboxyl, amino, thiol, and phenolic groups also can effectively chelate metal ions in the cytoplasm and reduce the toxic effects of heavy metals (Zhu et al., 2018). Furthermore, amino acids were observed to affect the synthesis and activity of some key enzymes, gene expression, and redox homeostasis (Chaffei-Haouari et al., 2009). In our experiments, 19 DEMs related to amino acid metabolism were significantly (*P* < 0.05) increased, and 8 pathways related to amino acid metabolism were up-graduated simultaneously as *T. pubescens* responds to Cd stress. It was reported that glutathione is a famous antioxidant protein response to the Cd presence as a cellular defense against ROS production (Sytar et al., 2013). Zoghlami et al. (2011) reported that asparagine, glutamine, and branched-chain amino acids (valine, isoleucine, phenylalanine, and tryptophan) significantly accumulated in the roots of tomatoes after Cd exposure. Proline was a key amino acid for Cd resistance because of its excellent features. Under such stress, proline was an osmotic pressure regulator against lipid peroxidation and a radical scavenger and stabilizer of macromolecules, and it can protect enzyme activity from inactivation and maintain the integrity of biofilm (Dhir et al., 2004). Cd stress would induce the increase of proline accumulation to alleviate Cd toxicity (Mahmood et al., 2014). Moreover, lysine metabolism could alleviate the toxicity of cadmium to fungi which were initially public in this study. Finally, we hypothesize that *T. pubescens* defends against Cd toxicity by rewiring nitrogen metabolism and enhancing energy metabolism to protect the cells against oxidative damage (Figure 7). NEAAs constitute components for redox homeostasis and the urea cycle and serve as important substrates for many nitrogenous compounds. NEAAs also can enter energy metabolism, and when necessary, they can be transferred to intermediates of glycolysis and TCA cycle by specific transaminases. Fungi under Cd stress were seen to upregulate NEAAs metabolism to defend against Cd toxicity to maintain intracellular redox homeostasis; on the other hand, AAs metabolized to fumarate or α-ketoglutarate (intermediates of TCA cycle) were found to enhance the energy metabolism to protect themselves against ROS accumulation, membrane disruption, and DNA replication damage.

## Conclusion

In summary, a heavy metal-resistant white-rot fungus *T. pubescens* was studied for its remarkable tolerance to cadmium. The maximum removal rate of *T. pubescens* was 53.13% in the concentration of Cd^2+^ at 10 mg/L. In the coculture with rice seedlings, it was illustrated that the colonization of *T. pubescens* was able to remarkably reduce the Cd toxicity and enable its protection of rice seedlings. Meanwhile, the fungus colonies were able to decrease Cd accumulation in rice tissue. Then, we performed transcriptomics and metabolomics to identify DEGs and DEMs related to *T. pubescens*-resistance mechanisms. It was noticed that the activation of nitrogen metabolism and energy metabolism resulted positively in the resistance of *T. pubescens* against Cd toxicity. To the best of our knowledge, this is the first integrative transcriptomic and metabolomic study for the genetic regulation and reprogramming of cell metabolism in response to Cd exposure by fungi. Our present results may provide a better understanding of the molecular resistance of *T. pubescens* in Cd stress. However, the specific mechanism of how nitrogen flow regulates the resistance of *T. pubescens* against Cd stress is not fully understood and thus awaits further elucidation in the future.

## Data availability statement

The data presented in this study are deposited in online repositories: The transcriptome datasets to the National Center for Biotechnology Information’s Sequence Read Archive with the accession number: PRJNA882141 and the UHPLC-MS/MS datasets to the MetaboLights database with the accession number: MTBLS5938.

## Author contributions

JL: conceptualization, methodology, software, resources, and writing—original draft. LW and XL: investigation. NE: supervision and resources. PF: writing—review and editing, supervision, project administration, and funding acquisition. All author: contributed to the article and approved the submitted version.

## Footnotes

1
https://www.ncbi.nlm.nih.gov/genome/?term=Trametes+pubescens

2
https://cloud.majorbio.com

3
http://bioconductor.org/packages/release/bioc/html/ropls.html

4
http://www.genome.jp/kegg/

## Abbreviations

*T. pubescens*: *Trametes pubescens*
Cd: cadmium
ROS: reactive oxygen species
MAPK: mitogen-activated protein kinase
α-KG: α-ketoglutarate
TCA: tricarboxylic acid
ATP: adenosine triphosphate
NADPH: reduced nicotinamide adenine dinucleotide phosphate.
PDA: potato dextrose agar
FTIR: Fourier transform infrared spectroscopy
SEM: scanning electron microscope
CONT: the control group
CONT(Cd): Cd treatment group
Tp (Cd): Cd plus *T. pubescens* group
NR: NCBI non-redundant protein sequences
Pfam: protein family
COG: Clusters of Orthologous Groups
GO: Gene Ontology
KEGG: Kyoto Encyclopedia of Genes and Genomes
DEGs: differentially expressed genes
UHPLC: ultra-high-performance chromatography
QC: quality control sample
RT: retention time
m/z: mass-to-charge ratio
OPLS-DA: orthogonal partial least squares discriminate analysis
VIP: variable importance in the projection
qRT-PCR: quantitative real-time PCR
ANOVA: one-way analysis of variance
Cd_L: *T. pubescens* in low concentration Cd stress
Cd_H: *T. pubescens* in high concentration Cd stress
CON: without Cd stress
DEMs: differentially expressed metabolites
PLS-DA: partial least squares discriminant analysis
HMDB: Human Metabolome Database
NEAAs: non-essential amino acids
GST: glutathione S-transferases.
