# Supplementary material for: A fungus (Trametes pubescens) resists cadmium toxicity by rewiring nitrogen metabolism and enhancing energy metabolism
Source: Front Microbiol. 2022 Nov 21;13:1040579. doi: 10.3389/fmicb.2022.1040579 (PMC9733723; doi:10.3389/fmicb.2022.1040579)
Supplement: Supplementary file 1 [file Data_Sheet_1.docx]

Supplementary Material

# Supplementary Data

Supplementary Material should be uploaded separately on submission. Please include any supplementary data, figures and/or tables. All supplementary files are deposited to FigShare for permanent storage and receive a DOI.

Supplementary material is not typeset so please ensure that all information is clearly presented, the appropriate caption is included in the file and not in the manuscript, and that the style conforms to the rest of the article. To avoid discrepancies between the published article and the supplementary material, please do not add the title, author list, affiliations or correspondence in the supplementary files.

# Supplementary Figures and Tables

## Supplementary Figures


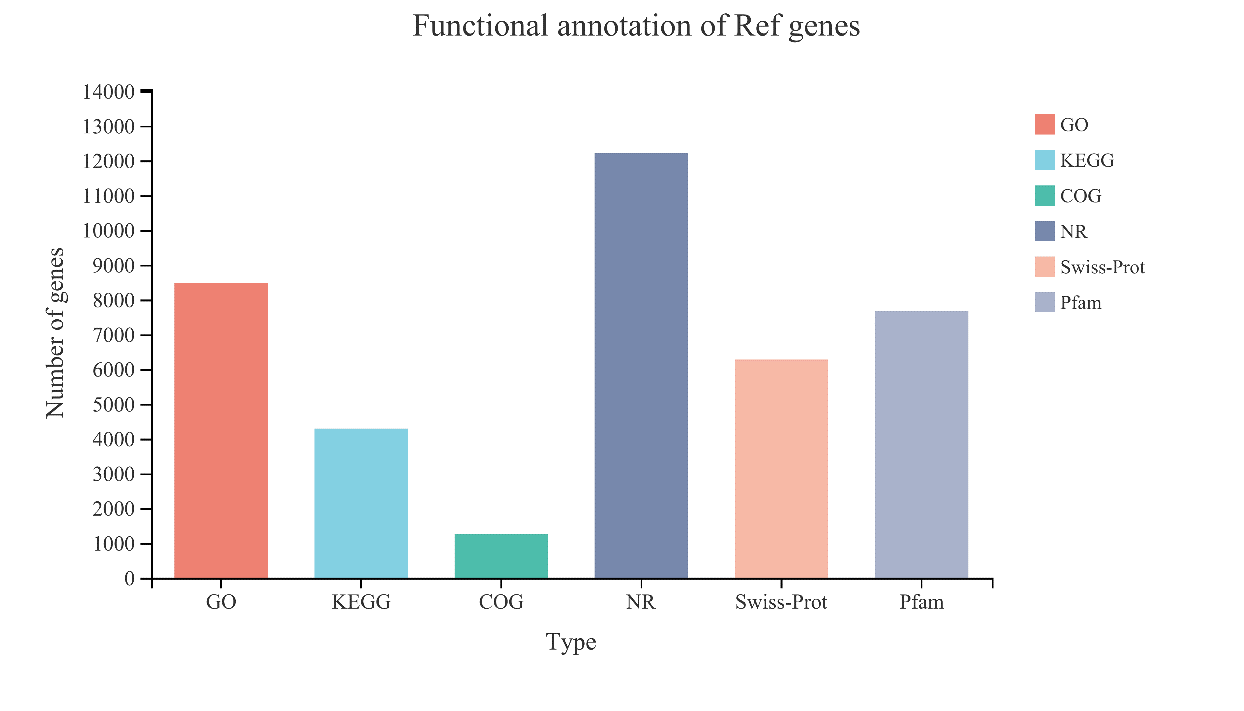


**Supplementary Figure 1.** Functional annotation of non-redundant transcripts in GO, KEGG, COG, NR, Swiss-Prot and Pfam databases. GO: Gene Ontology; KEGG: Kyoto Encyclopedia of Genes and Genomes; COG: Clusters of Orthologous Groups; NR: NCBI non-redundant protein sequences; Pfam: Protein family.


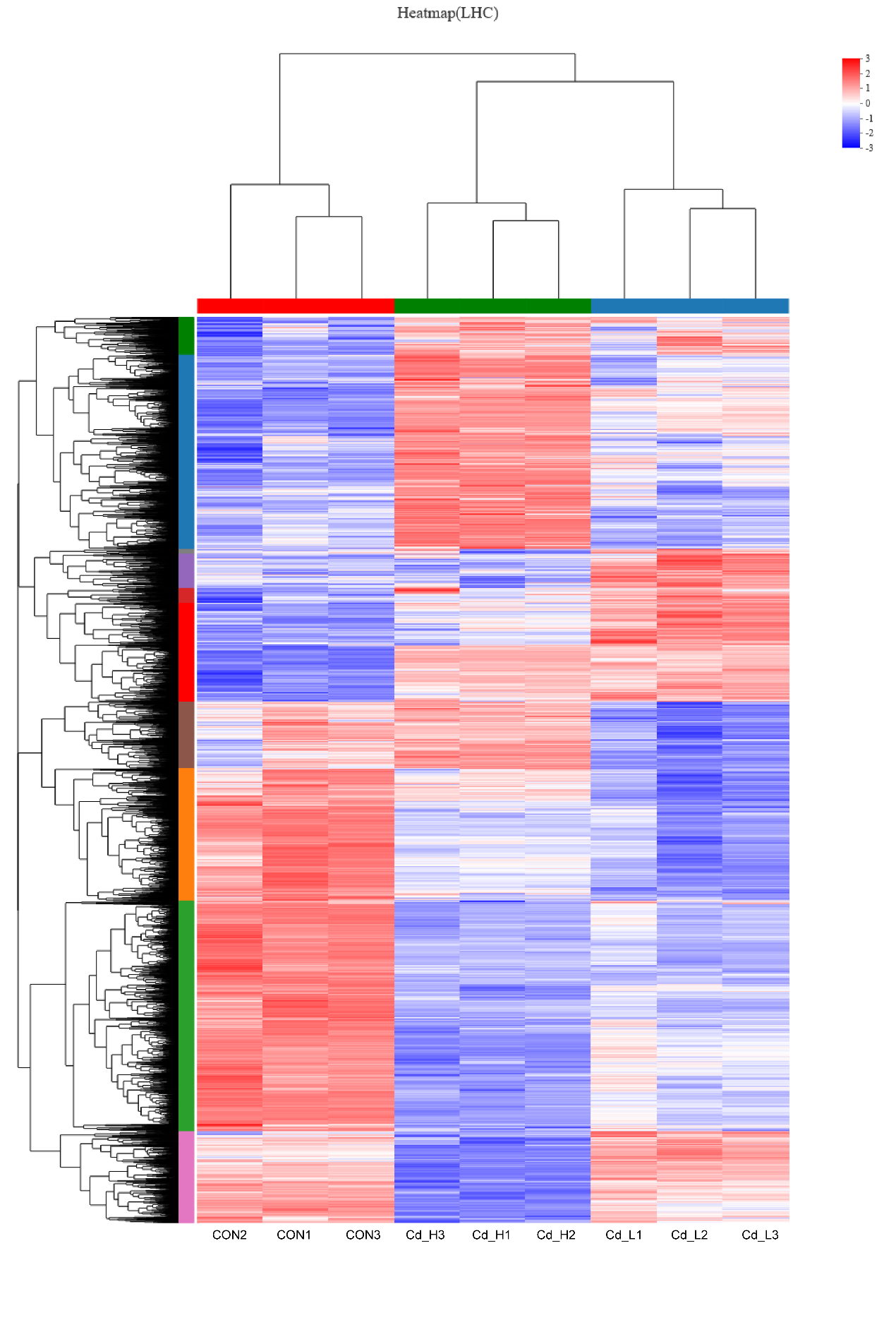


**Supplementary Figure 2.** Heat maps and sub-cluster trend map of DEGs. Cd: Cadmium; Cd_L: low-dose Cd treatment group; Cd_H: high-dose Cd treatment group; CON: no Cd treatment group; DEGs, differentially expressed genes.


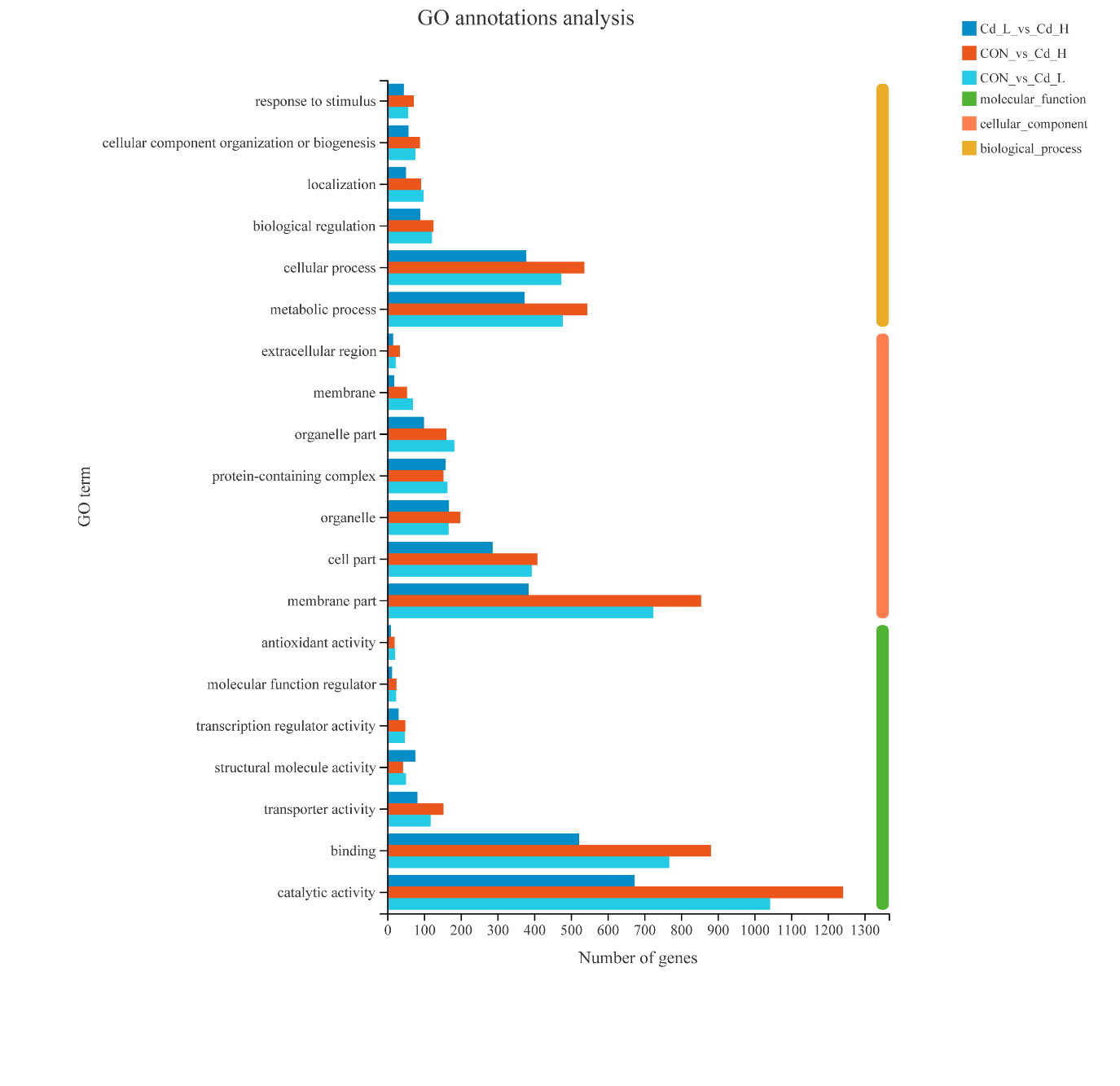


**Supplementary Figure 3.** Unigenes of *T. pubescens* under Cd stress annotated in GO databases. Cd: Cadmium; Cd_L: low-dose Cd treatment group; Cd_H: high-dose Cd treatment group; CON: no Cd treatment group; GO: Gene Ontology.


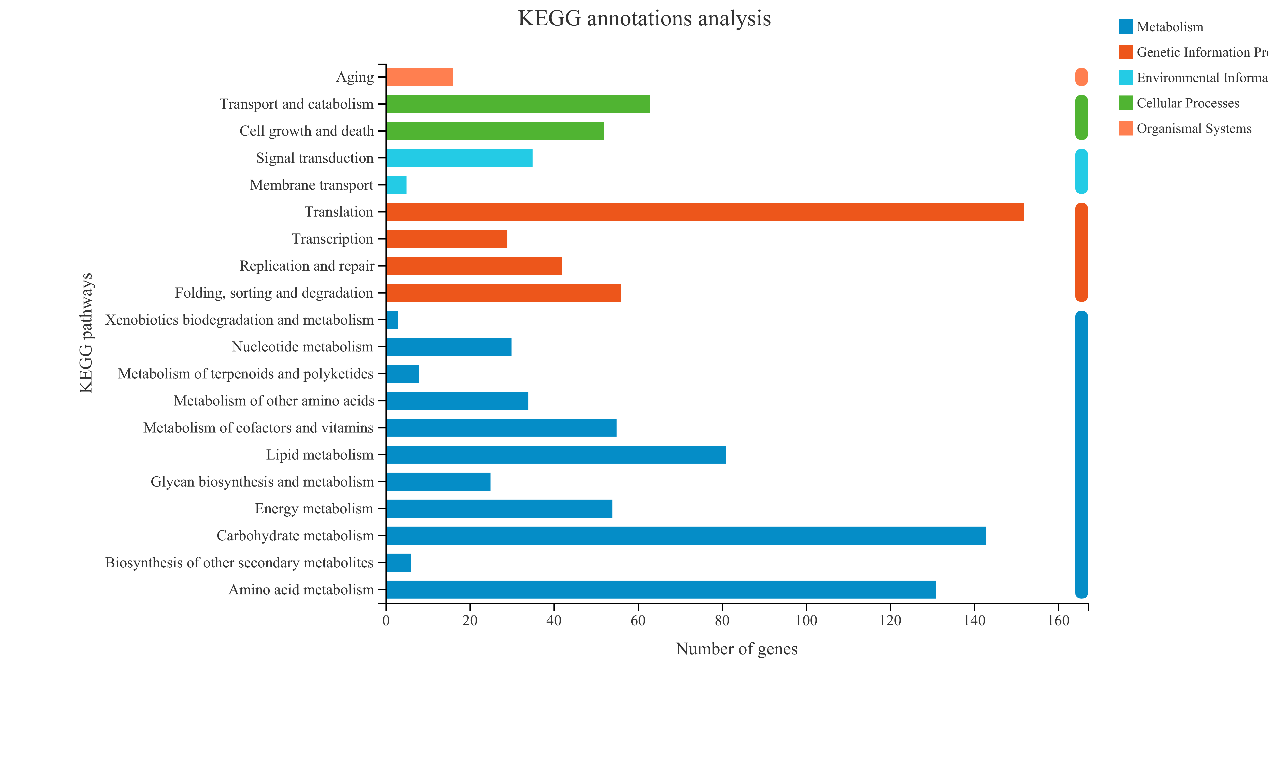


**Supplementary Figure 4.** Unigenes of *T. pubescens* under Cd stress annotated in KEGG databases. Cd_L: low-dose Cd treatment group; Cd_H: high-dose Cd treatment group; CON: no Cd treatment group; KEGG: Kyoto Encyclopedia of Genes and Genomes.


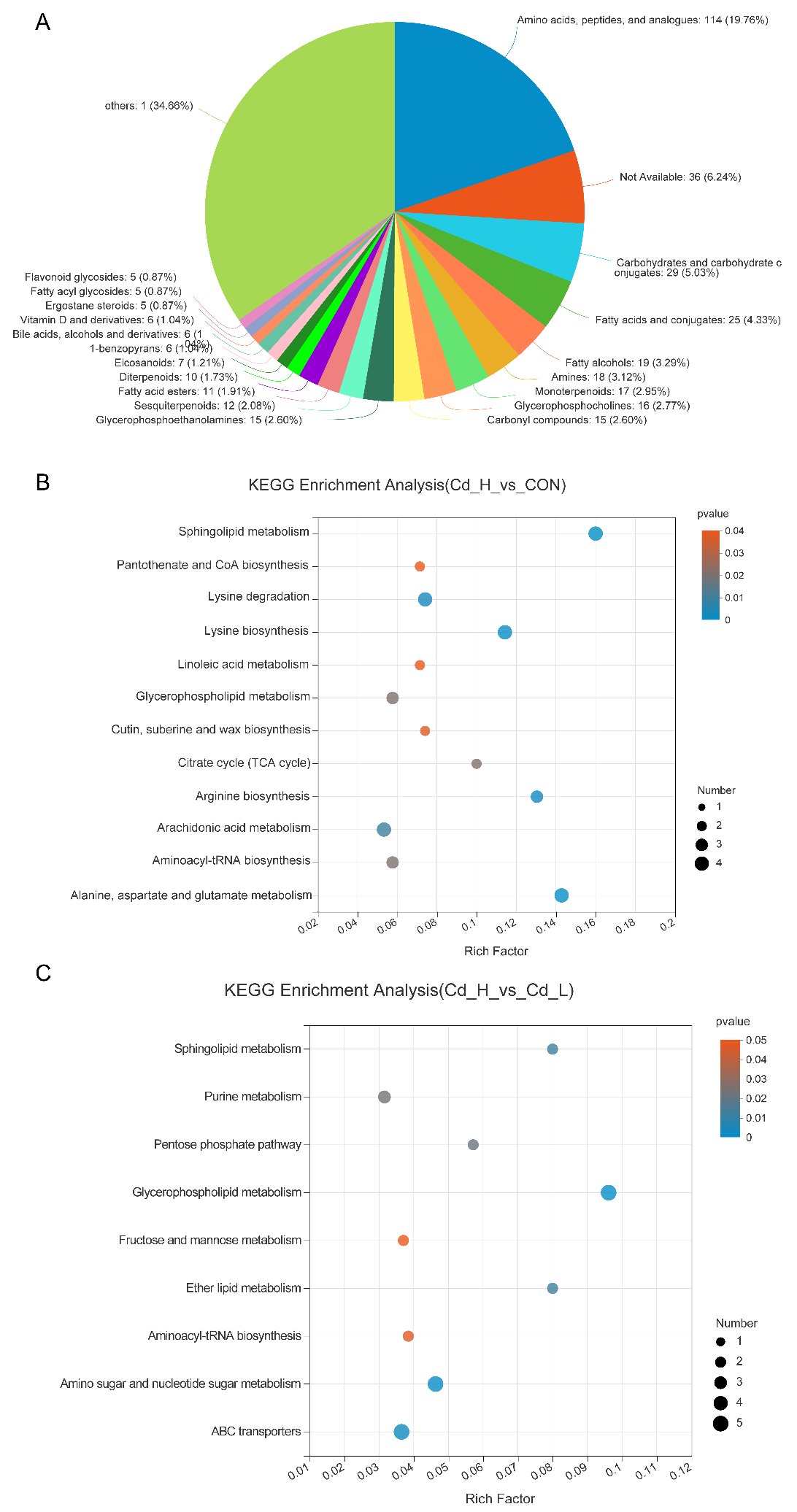


**Supplementary** Figure 5. Effects of Cd stress on *T. pubescens* metabonomic profiling by UHPLC-Q Exactive MS. (A) Pie chart of metabolite classification information comparing with HMDB 4.0 database. (B, C) KEGG enrichment analysis between groups. Cd: Cadmium; Cd_L: low-dose Cd treatment group; Cd_H: high-dose Cd treatment group; CON: no Cd treatment group; UHPLC: Ultra High Performance Liquid Chromatography; HMDB: Human Metabolome Database; KEGG: Kyoto Encyclopedia of Genes and Genomes.


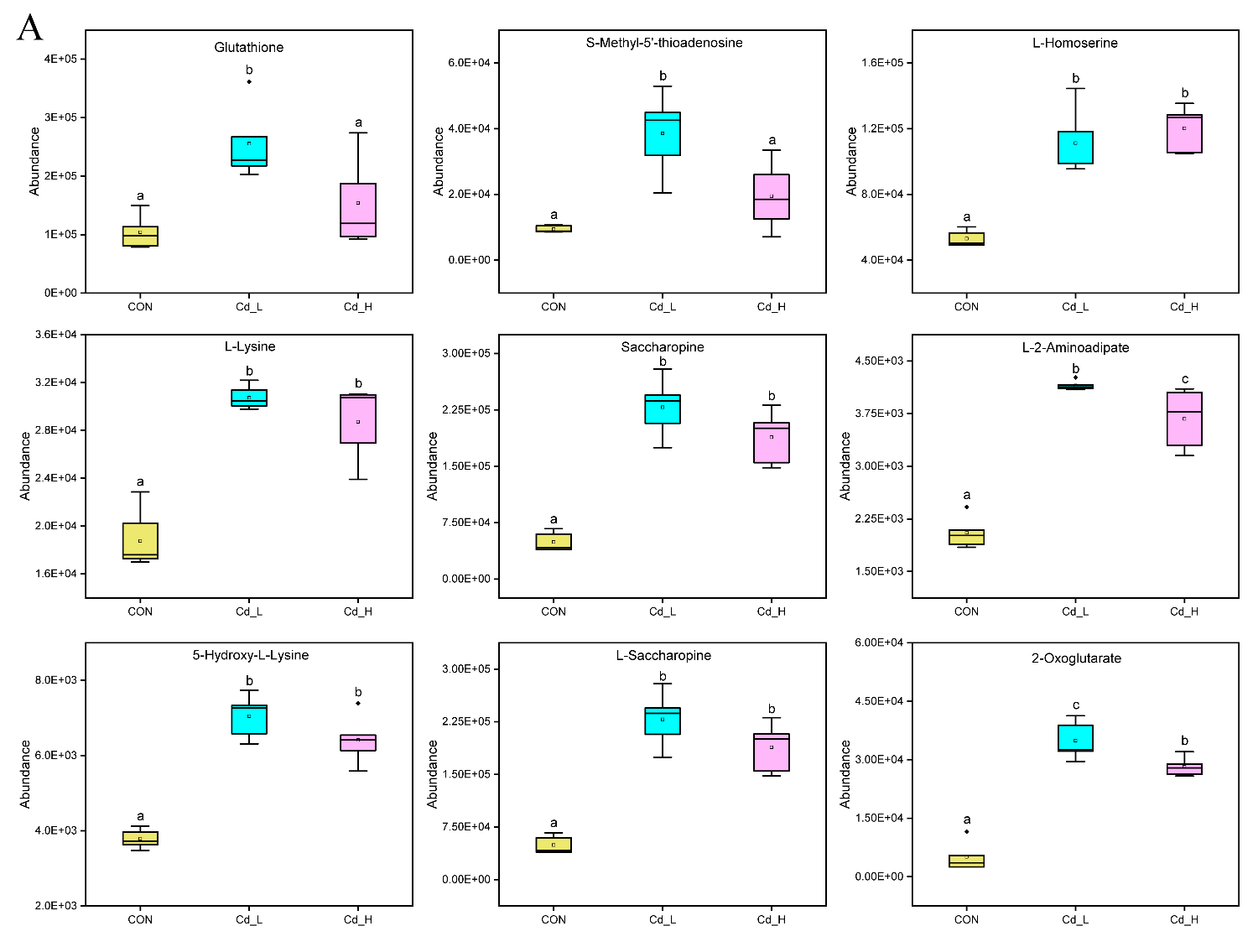

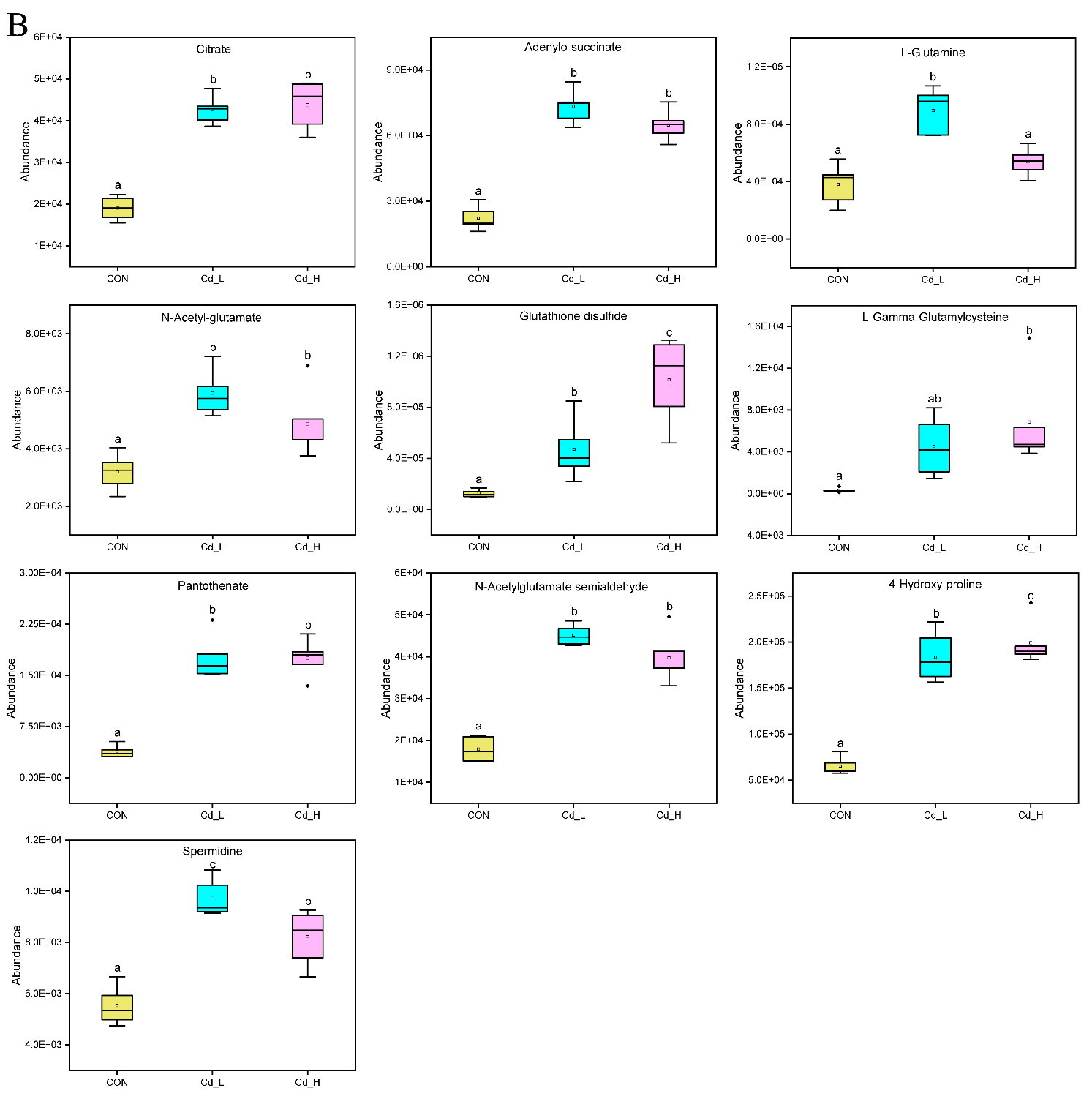


**Supplementary Figure 6.** Effect of Cd stress on amino acid metabolism-related metabolites content in *T. pubescens*. Different letters indicate significant differences between groups (p < 0.05). Cd: Cadmium; Cd_L: low-dose Cd treatment group; Cd_H: high-dose Cd treatment group; CON: no Cd treatment group.

## Supplementary Tables

**Supplementary** **Table 1.** The primer sequences used in this study.

| Gene | Sequence (5’ to 3’) | |
| --- | --- | --- |
| *48424-gpd* | Forward | TTCACTCTCTTCTCGCTTTCGTC |
|  | Reverse | ATCGTGTGGTTGGTAAGGTTGTC |
| *TRAPUB-12752* | Forward | ATTGACGCCCGGCATCTT |
|  | Reverse | GAACGCTTCCAAATCTGATACGA |
| *TRAPUB-14297* | Forward | GCCCTATGGCTGGCTTGCTA |
|  | Reverse | GTTCCGCCTGCGATACTACAA |
| *TRAPUB-8002* | Forward | TCCCAGTGACCTTGCTCCTATT |
|  | Reverse | ACCATAAGCAGATGCGAACGAA |
| *TRAPUB-13510* | Forward | GGCCCTGACTACAAGAAGAACCA |
|  | Reverse | TGGTCTTGTTGGAAGGCTGGA |
| *TRAPUB-12375* | Forward | TCTCGCTCGCTCTTTACCACC |
|  | Reverse | GCAGTCCCGATACAGGTTGAAAG |
| *TRAPUB-13778* | Forward | AGTTCAGTTACGGCGGTGGAG |
|  | Reverse | AGTGCCATTCCATATTTGCGAG |
| *TRAPUB-6157* | Forward | GGAATGATGAAACCGAGGACAG |
|  | Reverse | GAAGGTGCGTTGGGAGGAAA |
| *TRAPUB-10529* | Forward | CTTCATGTTCGCAATCGACTGG |
|  | Reverse | CGGGAGTAGTAGGCGGGTATGT |
