# Supplementary figures and images for: A fungus (Trametes pubescens) resists cadmium toxicity by rewiring nitrogen metabolism and enhancing energy metabolism
Source: Front Microbiol. 2022 Nov 21;13:1040579. doi: 10.3389/fmicb.2022.1040579 (PMC9733723; doi:10.3389/fmicb.2022.1040579)

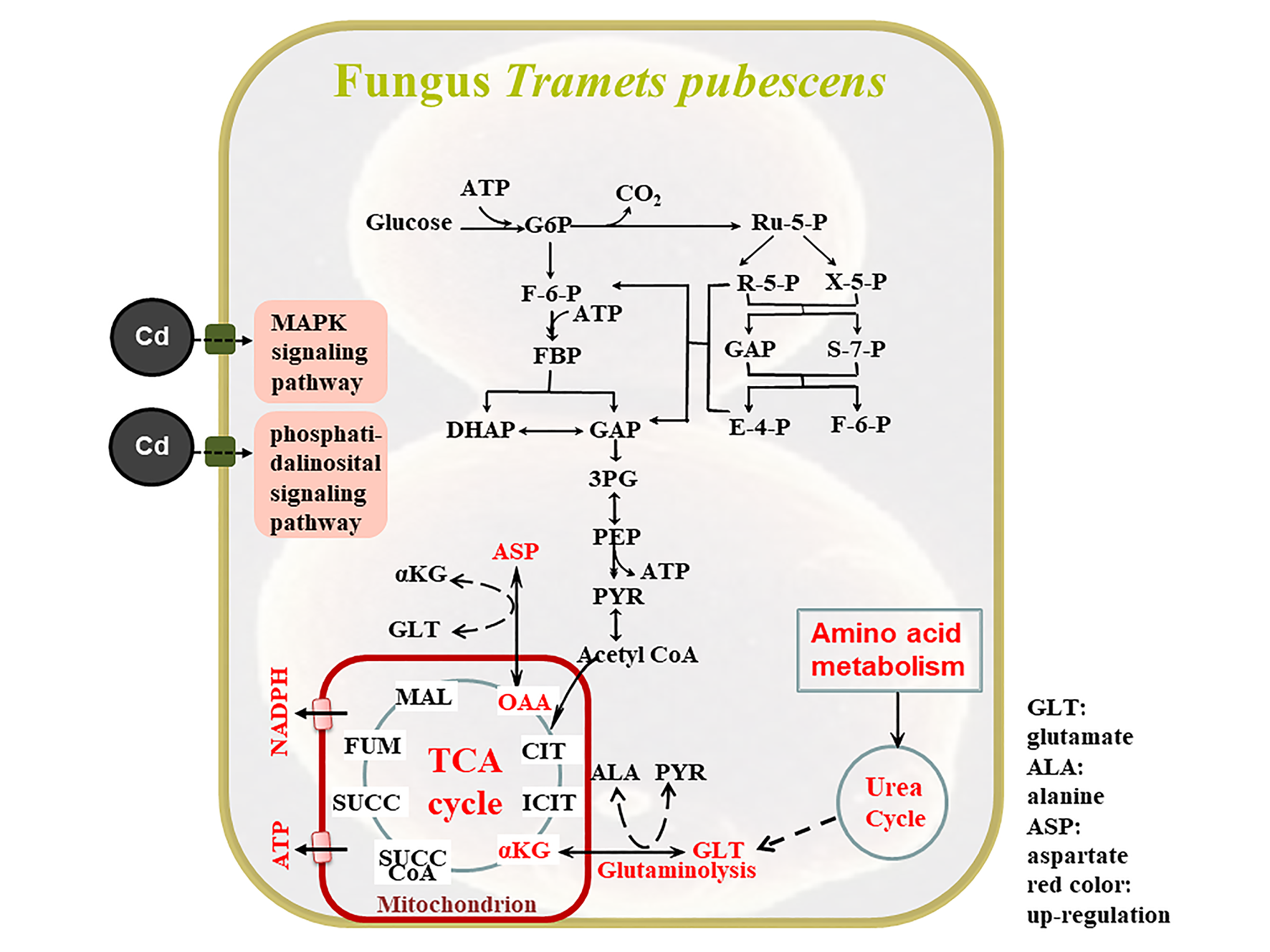

Supplement: Supplementary file 2 [file Image_1.TIF]
